# Late Total Cavopulmonary Connection in Adolescents and Adults: Narrative Review of Patient Selection and Outcomes

**DOI:** 10.3390/jcdd13070333

**Published:** 2026-07-15

**Authors:** Hichem Sakhi, Sébastien Hascoët, Emre Belli, Sarah Cohen

**Affiliations:** 1 Department of Cardiovascular Imaging, Hôpital Marie Lannelongue, Fondation Paris Saint Joseph, Université Paris-Saclay, 92350 Le Plessis Robinson, France; 2 Department of Congenital Heart Diseases, Centre de Référence Malformations Cardiaques Congénitales Complexes M3C, Hôpital Marie Lannelongue, Fondation Paris Saint Joseph, Université Paris-Saclay, 92350 Le Plessis Robinson, France

**Keywords:** adult congenital heart disease, total cavopulmonary connection, fontan procedure, late fontan completion, univentricular physiology, narrative review

## Abstract

**Background**: Although most patients with univentricular physiology undergo staged palliation during childhood, a small subset reaches adolescence or adulthood without complete cavopulmonary connection. The role of late total cavopulmonary connection (TCPC) in these patients remains poorly defined. This review summarizes the available evidence regarding TCPC surgery in adolescents and adults and discusses contemporary patient selection. **Methods**: A structured narrative review of PubMed/MEDLINE was conducted from database inception to December 2024. Studies reporting primary Fontan or TCPC completion in adolescents and adults were included, whereas studies exclusively addressing Fontan conversion were excluded. The review was conducted according to SANRA recommendations. **Results**: Sixteen studies reporting outcomes after late TCPC completion were identified. Most adult candidates presented with cyanosis, reduced functional capacity, previous palliation, and dominant left ventricular morphology. Surgical techniques evolved from atriopulmonary connections to lateral tunnel and extracardiac conduit TCPC. Early postoperative mortality ranged from 0% to 20%, with low cardiac output syndrome representing the leading cause of death. Reported survival at 5, 10, and 15 years ranged from 76 to 95%, 71–81%, and 66–79%, respectively. Preserved ventricular function, low pulmonary vascular resistance, and absence of significant atrioventricular valve regurgitation were consistently associated with better outcomes. **Conclusions**: Late TCPC surgery in carefully selected adults is feasible and may improve oxygenation and functional status. Contemporary decision-making should integrate anatomical, hemodynamic, functional, rhythm, and end-organ assessment while considering alternative strategies such as transplantation or conservative management.

## 1. Introduction

The prevalence of adults living with congenital heart disease (CHD) continues to increase, and adults now outnumber children in many developed healthcare systems [1,2]. Within this expanding population, patients with complex CHD and univentricular physiology represent a growing subgroup requiring highly specialized lifelong care [3]. Current adult congenital heart disease guidelines provide a framework for the management of Fontan circulation, but they offer limited specific guidance for adults who reach late adolescence or adulthood without complete cavopulmonary palliation [4,5,6]. The Fontan operation, first performed in 1968, was designed to channel systemic venous return directly into the pulmonary circulation without the need for a subpulmonary ventricle [7]. Since then, the technique has evolved from atriopulmonary connections to lateral tunnel and extracardiac total cavopulmonary connection (TCPC), with extracardiac conduits currently favored in many centers because of more favorable hemodynamics and rhythm outcomes [8,9]. Classical operability criteria emphasize young age, preserved ventricular function, low pulmonary vascular resistance, low ventricular filling pressures, competent atrioventricular valves, and unobstructed pulmonary arteries [10]. However, adult candidates differ from pediatric candidates because they have often been exposed for decades to cyanosis, ventricular volume overload, systemic-to-pulmonary shunts, arrhythmias, collateral circulation, and progressive end-organ injury. These factors may increase surgical risk and complicate the extrapolation of pediatric Fontan criteria to adults [11]. Nevertheless, selected adults with balanced univentricular physiology, preserved ventricular function, and favorable pulmonary vascular characteristics may still be considered for TCPC completion. The objective of this review is therefore to summarize the published experience of TCPC surgery in adult patients, including preoperative characteristics, indications, surgical strategies, early and late outcomes, and contemporary issues relevant to patient selection.

## 2. Materials and Methods

This narrative review was conducted according to the SANRA recommendations for the quality assessment of narrative reviews [12]. A structured search of PubMed/MEDLINE was performed from database inception to December 2024. The following search strategy was used: (“Fontan” OR “total cavopulmonary connection” OR “TCPC” OR “single ventricle”) AND (“adult” OR “adolescent” OR “grown-up congenital heart disease” OR “adult congenital heart disease”). Prospective studies, retrospective studies, and case series were considered eligible when they reported primary Fontan or TCPC completion in adolescents or adults with univentricular physiology. Studies including mixed adolescent and adult cohorts were retained when adult or late Fontan completion outcomes were clinically relevant. Non-English-language articles, studies with unavailable full text, isolated case reports, and studies exclusively reporting Fontan conversion from a previous atriopulmonary connection were excluded. The reference lists of included articles were manually screened to identify additional relevant studies. Given the heterogeneity of study design, anatomy, surgical technique, age at operation, and outcome reporting, results were synthesized narratively rather than through meta-analysis [13,14,15,16,17,18,19,20,21,22,23,24,25,26,27,28].

## 3. Results

### 3.1. Preoperative Patient Characteristics

This surgery was predominantly carried out during the second decade of the patients’ lives (Table 1). While procedures have been reported in individuals over 50 years old, such cases remain uncommon [17]. Even though these patients exhibit a variety of congenital heart defects (such as tricuspid atresia, double outlet right ventricles, mitral atresia, etc.), there is a predominance of single left univentricular hearts [14,15,17,18,22,23,24,25,26,28]. Considering the inferior hemodynamic performance of a single right ventricle, adult survivors with univentricular heart disease are more likely to possess a left ventricle [29,30,31]. Moreover, the occurrence of related abnormalities, such as vessel malposition or heterotaxy, can impact up to one-third of patients [17]. Nonetheless, most patients across all study series had undergone prior palliation before the TCPC procedure: either a systemic-pulmonary anastomosis (Blalock or other variants) or a partial cavopulmonary shunt (Glenn or other variants). In these studies, the median duration from palliation to TCPC was 6 to 7 years [22,23,24]. The occurrence of pulmonary artery banding is less common; however, it may have constituted up to 40% of patients in the series presented by Ono et al. [27]. Patients with univentricular heart disease who have never undergone surgery typically comprise 10 to 40% of the patient population [13,14,15,18].

Clinically, most patients are in NYHA stages 3–4. In the series by Burkhart et al., up to 87% of patients reported experiencing preoperative dyspnea at NYHA stage 3 or 4 [17,21]. On average, preoperative oxygen saturation levels typically range between 77% and 85% on room air [18,22,23]. Biologically, there is commonly a polycythemia resulting from cyanosis [22,27]. In these series, few cases of preoperative renal failure have been reported, although it is well-established that the risk of nephropathy increases during the second decade of life in cyanotic patients [32].

TCPC completion is generally considered in patients with preserved systemic ventricular systolic function, acceptable ventricular filling pressures, low pulmonary vascular resistance, and no more than mild-to-moderate atrioventricular valve regurgitation [10,33,34]. In adult candidates, these criteria should be interpreted cautiously because long-standing cyanosis and ventricular volume overload may impair diastolic function before systolic dysfunction becomes evident. In the series by Ono et al., up to 20% of patients had a functional single ventricle ejection fraction below 50% [16,27]. Similarly, moderate to severe atrioventricular valve regurgitation was uncommon overall, although one series reported at least moderate regurgitation in up to 15% of patients [19,23,27].

Preoperative cardiac catheterization remains central to decision-making. In most series, pulmonary vascular resistance was below 4 WU.m^2^ and mean pulmonary artery pressure was below 15 mmHg [17,18,20,23,24,27,28]. However, borderline hemodynamics were not rare: Podzolkov et al. and Gates et al. reported that 20% and 33% of patients, respectively, underwent surgery despite pulmonary hypertension [15,22]. In contemporary practice, assessment should also include transpulmonary gradient, ventricular end-diastolic pressure, pulmonary artery anatomy, collateral burden, and response to vasodilator testing when pulmonary vascular disease is suspected.

In terms of rhythm, the study by Fuji et al. found that up to 40% of patients experienced supraventricular rhythm disorders [20]. The occurrence of ventricular rhythm disorders during preoperative care appears to be much less common. The presence of conduction abnormalities in preoperative care necessitates the placement of epicardial electrodes during surgery.

### 3.2. Indications for Surgery

The decision to perform TCPC in adults who have not previously undergone complete palliation is mainly driven by cyanosis, reduced exercise capacity, and progressive symptoms [19,23]. Rhythm-related indications are less frequent than in Fontan conversion cohorts, in which arrhythmia surgery and conversion from atriopulmonary connections often represent a major component of management [34,35,36,37,38]. In adults, the indication should be individualized within an expert ACHD multidisciplinary team, balancing the expected improvement in oxygenation and ventricular unloading against the risk of Fontan failure, arrhythmia, thromboembolism, liver disease, and transplantation candidacy.

### 3.3. Surgical Techniques

**Figure 1:**
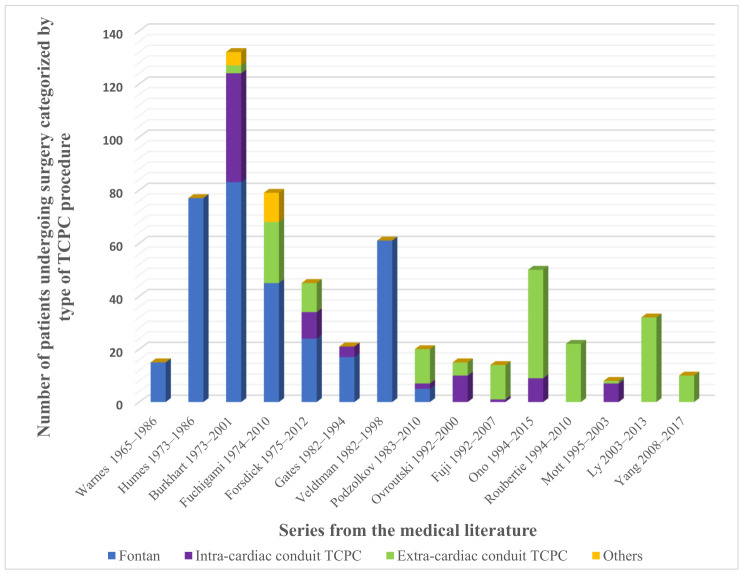
Types of surgeries reported in 15 medical literature series (with inclusion dates). TCPC: total cavopulmonary connection [13,14,15,16,17,18,19,20,22,23,24,25,26,27,28].

Since Fontan’s initial surgery in 1968, surgical techniques have progressively advanced. Atriopulmonary anastomosis was the predominant procedure in the 1970s and 1990s, but it gradually transitioned to intracardiac TCPC and now to TCPC with the incorporation of an extracardiac conduit [13,17,27] (Figure 1). Prior research in children has demonstrated that extracardiac TCPC enhances long-term survival and decreases the occurrence of arrhythmias [39].

Besides TCPC, additional surgical procedures can be combined with it, such as:

- Creating a fenestration, which varies significantly across different study series, ranging from 1.5% to 72% of patients [17,23]. The advantages of fenestration remain a subject of debate; however, it appears to lower the occurrence of pleural complications and shorten the duration of postoperative hospitalization [40].
- Rhythm-related procedures: The insertion of a pacemaker is necessary in 6% of surgeries to complete the procedure, and it is usually performed epicardially once the TCPC has been completed [20,27]. Concomitant ablation of rhythm disorders was carried out in 12.5% of patients in Ly et al.’s study [24]. In comparison, these procedures are considerably more common in Fontan conversions [41,42].
- Repair of the atrioventricular valve, the frequency of which varies across different study series but which could affect up to 40% of patients in Burkhart et al.’s study [17]. Atrioventricular valve replacements are less frequently performed.

The average duration of extracorporeal circulation varies, ranging from 115 to 221 min [19,26].

### 3.4. Short-Term Outcomes

The rate of mortality following surgery (within 30 days) is highly variable across different study series, ranging from 0 to 20% [14,19,24,28] (Table 2).

**Figure 2:**
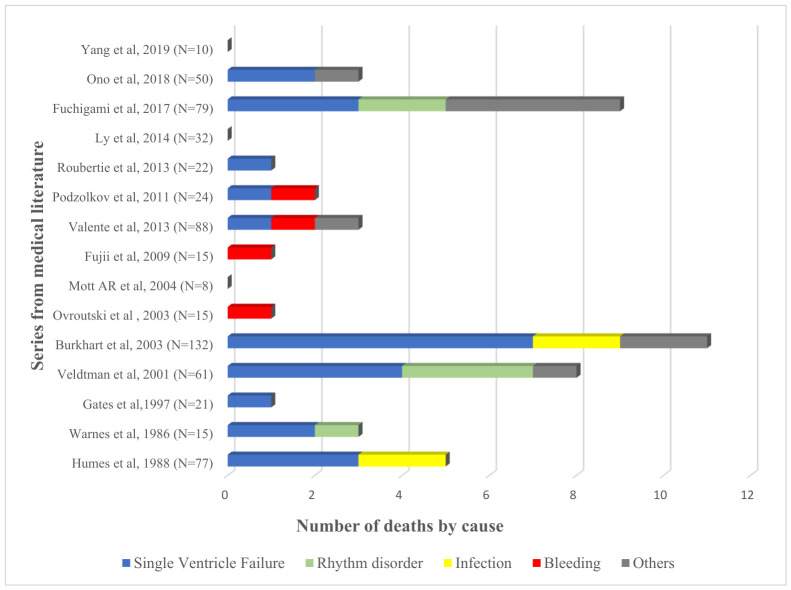
Number of deaths by cause within 30 days following surgery [13,14,15,16,17,18,19,20,21,22,23,24,26,27,28].

Among the causes of postoperative mortality reported, hemodynamic failure with low cardiac output of the systemic ventricle is clearly predominant and accounts for almost 50% of deaths in most study series. Other leading causes of death include infectious, rhythmic, or hemorrhagic complications. Less frequently, postoperative deaths result from increased postoperative pulmonary pressures, pulmonary embolism, or stroke [13,14,17,21,26] (Figure 2).

Factors that increase the risk of early mortality include a preoperative single ventricular ejection fraction below 45%, single ventricular end-diastolic pressures over 11 mmHg, at least moderate atrioventricular valve regurgitation before surgery, failure to meet more than two Choussat operability criteria, and preoperative or postoperative elevation in pulmonary vascular resistance greater than 4 WU [15,22,26].

The average length of hospital stay is also highly variable across studies, ranging from 8 to 46 days [19,20]. The average duration of mechanical ventilation is typically under 24 h [18,19,22]. Preoperative end-diastolic pressures of the single ventricle over 11 mmHg appear to prolong the duration of mechanical ventilation [18]. One of the primary postoperative complications is the persistence of prolonged pleural effusions, which necessitate continued use of pleural drainage [17]. Advanced age, decreased preoperative single ventricular ejection fraction, and longer duration of extracorporeal circulation are associated with an increased likelihood of postoperative pleural effusions [24]. In one of the study series, the presence of ascites requiring puncture was reported in 16% of patients [26].

Up to 25% of patients have been reported to experience supraventricular rhythm disorders [22]. Ventricular and conduction rhythm disturbances are less common in the immediate postoperative period. Extracardiac TCPC yields better rhythm-related outcomes than intracardiac TCPC, as in children [18]. The requirement for immediate postoperative pacemaker implantation was reported in only three patients across two study series [18,22].

Postoperative heart failure is observed in less than 10% of patients in most study series [13,14,16,17]. The presence of a tricuspid valve as an atrioventricular valve and preoperative pulmonary artery banding are risk factors for postoperative single ventricular dysfunction [26].

Hemorrhagic complications necessitating hemostasis intervention may have occurred in up to 8% of patients in the Burkhart et al. study [17].

Less frequent complications include pneumonia, sepsis, stroke, pulmonary embolism, mediastinitis, and renal failure requiring dialysis.

Upon hospital discharge, arterial oxygen saturation is significantly higher than preoperatively, with an average of 93% in ambient air [17,21,23]. Patients who have undergone TCPC with an extracardiac tube generally exhibit better arterial oxygen saturation than those who received a TCPC with an intracardiac tube [18]. The single ventricular ejection fraction may experience a transient decrease in the postoperative period [22]. Average left atrial pressures at hospital discharge range from 7 to 10 mmHg [18,20,22].

### 3.5. Long-Term Outcomes

Reported mortality rates at 5, 10, and 15 years in four studies are 76–95%, 71–81%, and 66–79%, respectively [15,16,17,21,25] (Table 2).

**Figure 3:**
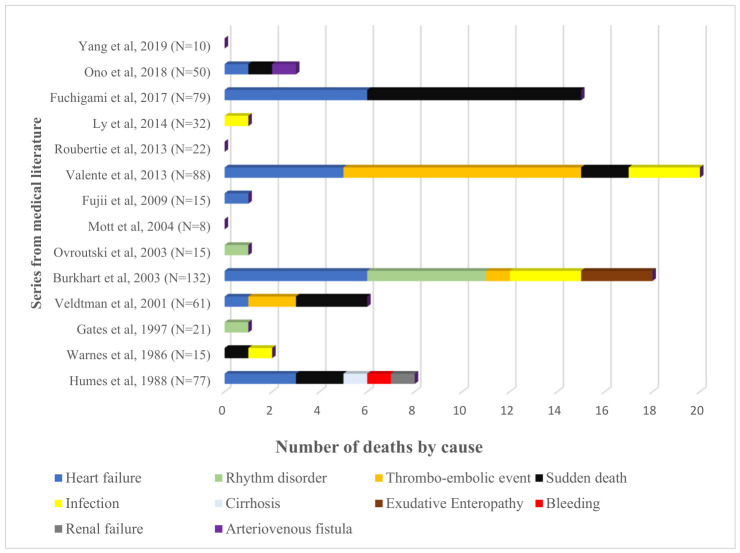
Number of deaths by cause beyond 30 days following surgery [13,14,15,16,17,18,19,20,21,23,24,26,27,28].

Heart failure appears to be the primary cause of late deaths (occurring more than 30 days after surgery). Rhythm disorders, sepsis, and thromboembolic events such as pulmonary embolism and stroke are also frequent. Less commonly, deaths may result from exudative enteropathy, cirrhosis, kidney failure, hypoxia from arteriovenous fistula, and hemorrhagic events [13,14,15,16,17,18,20,21,24,27] (Figure 3).

There is also a relatively high incidence of sudden deaths, which can account for up to 47% of deaths in the Fuchigami et al. study [26]. In the same study, half of the patients who died of sudden death were being monitored for a postoperative rhythm disorder.

Risk factors for long-term mortality include age over 30 years, male sex, preoperative mean pulmonary arterial pressure over 15 mmHg, postoperative right atrial pressure over 20 mmHg, and postoperative recovery for hemorrhage [16,17,20].

Up to one-third of patients can experience heart failure as a long-term complication [16]. Supraventricular rhythm disorders are prevalent and can affect up to 25% of patients, while conduction disorders are rare in the immediate postoperative period but become more common during long-term follow-up. In such cases, the implantation of a pacemaker may be necessary [13,16,17]. According to the Burkhart et al. study, nearly 15% of surviving patients required the implantation of a pacemaker [17]. Similarly, ventricular rhythm disorders are less frequent but still significant in the long-term follow-up. The Warnes et al. series reported a ventricular rhythm abnormality in 10% of patients during follow-up [14].

According to the study by Veldtman et al., thromboembolic complications, such as pulmonary embolism or stroke, are relatively common, with a reported incidence of 20% among patients [16]. However, the optimal anticoagulation strategy for primary prevention in TCPC patients remains controversial [43].

Other possible complications may include obstruction in the circuit, protein-losing enteropathy, cirrhosis, arteriovenous fistulas, or renal failure. According to the study by Veldtman et al., the rate of re-operation after TCPC can be as high as 28% at a median of 11 years post-surgery, with a range of 2–18 years [16]. Late re-interventions can be required due to various causes such as an obstruction in the Fontan circuit, rhythm disorders necessitating the conversion of a “classic” Fontan, conductive disorders necessitating the implantation of a pacemaker, or repair/replacement of an atrioventricular valve [16,17].

Most surviving patients who underwent TCPC show clinical improvement and are classified as NYHA stage 1 or 2. The average arterial oxygen saturation remains around 92% [15,16,17,18,19,20,21]. Two studies have examined the peak oxygen consumption of postoperative patients and reported similar mean values of around 22–23 mL/min/kg [18,27]. In the Humes et al. study, it was found that 93% of the 61 surviving patients could return to work [13].

## 4. Discussion

### 4.1. Impact of Age at the Time of TCPC

The impact of age at TCPC completion remains difficult to isolate because older patients differ from younger candidates in anatomy, previous palliation, duration of cyanosis, ventricular loading conditions, arrhythmia burden, and end-organ exposure. Humes et al. reported a mortality rate of 5% among patients older than 18 years, whereas the same center reported 17% mortality in a broader age cohort during the same period. Valente et al. suggested that age at totalization was not an independent risk factor [13,21]. In contrast, Burkhart et al. identified age over 30 years as a predictor of mortality [17]. These divergent findings likely reflect selection bias and era effects rather than age alone.

According to Ono et al., short-term outcomes may be comparable across age groups, but older patients tend to experience more impaired ventricular function and lower exercise capacity during long-term follow-up [27]. Pediatric and adolescent studies also suggest that later Fontan completion is associated with poorer exercise performance [44,45]. Overall, age should not be regarded as an isolated contraindication, but as a marker of cumulative exposure to cyanosis, ventricular volume overload, abnormal pulmonary blood flow, and end-organ injury.

### 4.2. Comparison of the Outcome of Patients with TCPC Versus Non Operative or Alternative Strategies

Patients with tricuspid atresia who have undergone Fontan surgery have been shown to have better exercise capacity than those without surgery or with simple palliation, as reported by Warnes et al. [14].

Veldtman et al. reported that Fontan surgery patients have better single ventricular ejection fraction, a more favorable NYHA class, and a lower incidence of supraventricular rhythm disorders compared to non-operated patients [16].

However, Forsdick et al. emphasized that survival alone may underestimate the long-term burden of late Fontan completion, as survivors remained exposed to Fontan failure, arrhythmias, transplantation, and other major adverse events during follow-up [25].

The arrival into adulthood of patients with an equivalent univentricular heart who have never undergone surgery or palliation appears to be uncommon. Poterucha et al. reported on the follow-up of twenty-four patients over thirty years at the Mayo Clinic, most of whom had a double outlet left ventricle with pulmonary stenosis [46]. Although likely a minority, it is understandable that there remains a population of patients with a “balanced” univentricular heart for whom the benefit of a Fontan procedure is uncertain.

### 4.3. Contemporary Assessment and Patient Selection

Adult candidates for late TCPC require a broader assessment than historical pediatric Fontan criteria. Echocardiography remains essential for evaluating ventricular morphology, systolic function, atrioventricular valve regurgitation, outflow obstruction, and venous pathways. Cardiac magnetic resonance and computed tomography provide complementary information on ventricular volumes, pulmonary artery anatomy, systemic and venous collaterals, thrombus, and surgical planning. Cardiopulmonary exercise testing is particularly useful to quantify functional limitation and to establish a baseline for postoperative follow-up. Invasive hemodynamic assessment should document mean pulmonary artery pressure, pulmonary vascular resistance, ventricular end-diastolic pressure, transpulmonary gradient, and pulmonary artery anatomy. In contemporary ACHD practice, end-organ assessment should also include renal function, liver imaging and fibrosis markers, coagulation profile, arrhythmia evaluation, and assessment for protein-losing enteropathy or lymphatic complications [5,6].

### 4.4. Long-Term Fontan-Related Complications in Adult Candidates

The historical series summarized in this review mainly focused on mortality, functional class, arrhythmias, and reinterventions. Contemporary evaluation must also account for Fontan-associated liver disease, renal dysfunction, thromboembolic risk, lymphatic failure, plastic bronchitis, protein-losing enteropathy, and advanced heart failure [47,48]. These complications may already be present before TCPC completion in chronically cyanotic or partially palliated adults and may influence both operative risk and the choice between TCPC, partial cavopulmonary palliation, conservative management, or transplantation. Therefore, late TCPC should be considered not only as a surgical procedure but as part of a lifelong Fontan pathway requiring structured surveillance.

### 4.5. Limitations

This review has several limitations. First, most available studies are retrospective, single-center series with small sample sizes and important era effects. Second, included patients were heterogeneous with respect to anatomy, ventricular morphology, previous palliation, age at operation, surgical technique, and follow-up duration. Third, the definition and reporting of postoperative complications were inconsistent across studies. Fourth, several series were performed before contemporary imaging, electrophysiology, liver surveillance, lymphatic imaging, and advanced heart failure strategies became routine. Moreover, this article is a narrative rather than systematic review, and the heterogeneity of available data precluded meta-analysis. As this work was designed as a narrative review in accordance with SANRA recommendations, the literature search was not conducted following PRISMA methodology, and no formal study selection process or methodological quality assessment was performed. Consequently, despite a structured search strategy and manual screening of reference lists, the possibility that some relevant studies were not identified cannot be completely excluded. In addition, the included studies exhibited substantial heterogeneity in patient characteristics, surgical strategies, and follow-up duration, which may have influenced the reported outcomes and limited direct comparisons across studies. Finally, restricting the literature search to a single database may have resulted in the omission of some eligible studies.

### 4.6. Perspective

Symptomatic adults with univentricular physiology may be considered for several strategies, including optimized medical treatment, systemic-to-pulmonary shunt revision, bidirectional cavopulmonary connection [22,49], TCPC completion, heart transplantation [50,51,52] or conservative management.

The optimal strategy depends on anatomy, pulmonary vascular status, ventricular function, atrioventricular valve competence, arrhythmia burden, end-organ involvement, and patient goals. Future studies should aim to develop adult-specific risk models for late TCPC completion. Multicenter registries are particularly needed because randomized trials are unlikely in this rare population. Emerging tools such as 4D-flow MRI, computational fluid dynamics, advanced lymphatic imaging, biomarkers, and structured Fontan surveillance programs may help refine patient selection and predict postoperative Fontan performance [53].

Because of the complexity of these patients, evaluation for late TCPC should ideally be performed within experienced multidisciplinary ACHD centers. A collaborative approach involving congenital cardiologists, congenital cardiac surgeons, imaging specialists, electrophysiologists, heart failure specialists, hepatologists, and transplant teams is essential to optimize patient selection and long-term outcomes.

## 5. Conclusions

Adults with univentricular physiology who remain unpalliated or partially palliated are uncommon but continue to be encountered in specialized ACHD centers. The available literature suggests that late TCPC completion can be feasible and may provide meaningful improvement in oxygenation and functional status in carefully selected patients. Favorable candidates generally have preserved ventricular function, low pulmonary vascular resistance, acceptable ventricular filling pressures, no severe atrioventricular valve regurgitation, suitable pulmonary artery anatomy, and limited end-organ disease. However, the evidence remains limited and heterogeneous, and the decision to proceed with TCPC must be individualized within an expert multidisciplinary team. Prospective multicenter registries and comparative studies are needed to better identify patients who benefit from late TCPC and those for whom alternative strategies, including transplantation or conservative management, may be more appropriate.

## Figures and Tables

**Table 1:** Summary of key patient characteristics in 16 single-center studies.

Articles	Number of Patients	Patient Inclusion Dates	Mean or Median Age	Age Range	Women, *n* (%)	Patients with a Right Single Ventricle, *n* (%)	Patients with Prior Palliation, *n* (%)	Abnormal Ejection Fraction, *n* (%)	Mean mPAP, mmhg	Mean PVR, WU.m^2^	Mean LVEDP, mmhg
Humes et al. [13]	77	1973–1986	24	18–42	31 (40%)	29 (37%)	64 (83%)	ND	ND	1.8	14
Warnes et al. [14]	15	1965–1986	16	9–25	8 (53%)	0 (0%)	13 (86%)	ND	ND	ND	ND
Gates et al. [15]	21	1982–1994	27	18–40	9 (43%)	3 (14%)	13 (61%)	6 (29%)	ND	ND	ND
Veldtman et al. [16]	61	1982–1998	36	18–47	29 (48%)	ND	49 (80%)	3 (5%)	ND	ND	ND
Burkhart et al. [17]	132	1973–2001	23	18–53	58 (44%)	31 (23%)	118 (89%)	ND	14	ND	ND
Ovroutski et al. [18]	15	1992–2000	26	16–38	ND	4 (26%)	7 (46%)	ND	12	ND	7
Mott AR et al. [19]	8	1995–2003	23	18–41	ND	3 (37%)	6 (75%)	2 (25%)	15	1.5	9
Fuji et al. [20]	15	1992–2007	29	18–52	7 (47%)	6 (40%)	12 (80%)	ND	11	2.4	ND
Valente et al. [21]	88	1973–2007	ND	15–40	43 (49%)	16 (18%)	78 (88%)	ND	ND	ND	ND
Podzolkov et al. [22]	24	1983–2010	24	18–38	ND	5 (20%)	18 (75%)	ND	12	ND	10
Roubertie et al. [23]	22	1994–2010	21	ND	9 (41%)	4 (19%)	22 (100%)	2 (9%)	12	1.8	9
Ly et al. [24]	32	2003–2013	24	18–47	17 (53%)	5 (13%)	25 (78%)	ND	11	1.8	8
Forsdick et al. [25]	45	1975–2012	18.3	16–21	20 (44%)	9 (20%)	29 (64%)	ND	ND	ND	ND
Fuchigami et al. [26]	79	1974–2010	20	15–32	41 (52%)	20 (25%)	ND	ND	ND	ND	ND
Ono M et al. [27]	50	1994–2015	13	ND	32 (64%)	27 (54%)	27 (54%)	10 (20%)	12	ND	ND
Yang et al. [28]	10	2008–2017	26	18–31	ND	ND	ND	ND	14	ND	ND

LVEDP: left ventricular end-diastolic pressure, mPAP: mean pulmonary artery pressure, PVR: pulmonary vascular resistance, ND: no data.

**Table 2:** Summary of the different series regarding the type of surgery and short- and long-term mortality.

Articles	Number of Patients for Each Type of Surgery Performed	Deaths Within 30 Days, *n* (%)	Survival at 1 Year, %	Survival at 5 Years, %	Survival at 10 Years, %	Survival at 15 Years, %
Humes et al. [13]	77 Fontan	5 (6.4%)	ND	ND	ND	ND
Warnes et al. [14]	15 Fontan	3 (20.0%)	66%	ND	ND	ND
Gates et al. [15]	17 Fontan 4 IC TCPC	1 (4.8%)	ND	95%	81%	ND
Veldtman et al. [16]	61 Fontan	8 (13.1%)	80%	76%	72%	67%
Burkhart et al. [17]	83 Fontan 41 IC TCPC 3 EC TCPC 5 Others	11 (8.3%)	ND	89%, 95% CI (84, 95)	75%, 95% CI (67, 84)	68%, 95% CI (70, 91)
Ovroutski et al. [18]	10 IC TCPC 5 EC TCPC	1 (6.6%)	93%	ND	ND	ND
Mott AR et al. [19]	7 IC TCPC 1 EC TCPC	0 (0.0%)	100%	ND	ND	ND
Fuji et al. [20]	1 IC TCPC 13 EC TCPC	1 (6.6%)	ND	ND	ND	ND
Valente et al. [21]	ND	3 (3.4%)	ND	83%	71%	66%
Podzolkov et al. [22]	5 Fontan 2 IC TCPC 17 EC TCPC	2 (8.3%)	ND	ND	ND	ND
Roubertie et al. [23]	22 EC TCPC	1 (4.5%)	ND	ND	ND	ND
Ly et al. [24]	32 EC TCPC	0 (0.0%)	ND	ND	ND	ND
Forsdick et al. [25]	24 Fontan 10 EC TCPC 11 IC TCPC	6 (13%)	ND	79% 95% CI (64, 89)	79% 95% CI (64, 89)	79% 95% CI (64, 89)
Fuchigami et al. [26]	45 Fontan 11 Bjork 23 EC TCPC	9 (11.3%)	ND	79% *	77% *	73% *
Ono M et al. [27]	9 IC TCPC 41 EC TCPC	3 (6.0%)	ND	ND	ND	ND
Yang et al. [28]	10 EC TCPC	0 (0.0%)	ND	ND	ND	ND

EC: extracardiac conduit, IC: intra-cardiac conduit, ND: no data, TCPC: total cavopulmonary connection; * Freedom from death or Fontan Takedown. CI, confidence interval. The 95% confidence interval is reported only when provided in the original publication.

## Data Availability

No new data were created or analyzed in this study. Data sharing is not applicable to this article.

## Abbreviations

The following abbreviations are used in this manuscript:

ACHD	Adult Congenital Heart Disease
CHD	Congenital Heart Disease
MRI	Magnetic Resonance Imaging
NYHA	New York Heart Association
SANRA	Scale for the Assessment of Narrative Review Articles
TCPC	Total Cavopulmonary Connection
WU	Wood Unit
